# Relief in Belgium

**Published:** 1916-08-12

**Authors:** 


### RELIEF IN BELGIUM.

The first annual report of the Commission for Relief in Belgium covers the twelve months extending from November 1, 1914, to October 31, 1915. The work described may roughly be separated into two parts, the first of which concerns the revictualment of the entire population of the greater part of Belgium and of Northern France, with the exception of a zone adjoining the firing line, and the second the distribution of relief to the destitute part of that population. Although the whole of the work of the Commission is benevolent in character, the larger part is a series of huge operations of a commercial nature : buying, shipping, and selling, and, in fact, making profits which, together with the gifts of charitable people, provide the necessary means to carry out the purely eleemosynary portion of the general scheme, vast in itself, though smaller as compared with the great operation of revictualment.

Industry becoming paralysed by a blockade which prevents the entry of raw materials upon which the factories are dependent, all means of transit being largely taken over for military purposes, and credit being practically extinguished, a population of nine to ten millions were in imminent danger of starvation. Large numbers became destitute, and larger, although having money, could not buy commodities which were no longer for sale. Hence the need for the mission of the Relief Commission, the work of which is necessarily divided into two parts—namely, provision and distribution. The provision is in the hands of the Commission for Relief in Belgium, a large neutral body, chiefly American, and the distribution is entrusted to two native committees, who are assisted by about 4,000 communal committees. During the year covered by the report 988,852 metric tons of goods were handled, nearly all foodstuffs; of these 890,552 tons were bought, and 98,300 given in kind. For transport a regular service of cargo ships was required, which carried 186 full and 308 part cargoes; the food nearly all came from North and South America, and was unshipped at Rotterdam, being conveyed from that port mostly in canal boats to 156 warehouse terminals, later to be distributed through 4,657 communal warehouses. The imports were confined as nearly as possible to seven principal commodities. The normal net annual imports of human food are 1,690,000 metric tons, and of fodder 1,350,000 tons; the imports by the Commission for one year were 640,934 tons of food and 102,815 of fodder. Elaborate checks were provided to ensure that no part of this large supply miscarried, and the report shows in tabular form how every ton reached the proper destination. In addition, every means were taken to safeguard primary economy and efficiency in purchase and transport.

The total value of purchases and gifts amounted to £12,674,848, the freights cost £2,345,020, the goods so obtained sold for £15,917,007. The total cost of administration was £101,994, or less than three-quarters of 1 per cent. of the turnover. On the benevolent side between two and a half and three million people who could not hope to make any payment in return were relieved at a cost of £3,493,637. The National Committee for Relief in Belgium (Trafalgar Buildings, Trafalgar Square, W.) is the body which collects and receives on behalf of the neutral Commission all contributions throughout the British Empire; this committee has already paid over to the Commission £1,600,000 for the purchase of food for the destitute Belgians in that part of their country occupied by **Germany.**

